# The impact of expertise in olfaction

**DOI:** 10.3389/fpsyg.2013.00928

**Published:** 2013-12-13

**Authors:** Jean-Pierre Royet, Jane Plailly, Anne-Lise Saive, Alexandra Veyrac, Chantal Delon-Martin

**Affiliations:** ^1^Olfaction: From Coding to Memory Team, Centre de Recherche en Neurosciences de Lyon, CNRS UMR 5292, INSERM U1028, Université Lyon 1Lyon, France; ^2^INSERM, U836, NeuroImagerie Fonctionnelle et Perfusion CerebraleGrenoble, France; ^3^Université Joseph Fourier, Grenoble Institut des NeurosciencesGrenoble, France

**Keywords:** odor expert, perfumer, oenologist, mental imagery, perceptual learning, functional and structural reorganization, brain plasticity, neurogenesis

## Abstract

Olfactory expertise remains poorly understood, most likely because experts in odor, such as perfumers, sommeliers, and oenologists, are much rarer than experts in other modalities, such as musicians or sportsmen. In this review, we address the specificities of odor expertise in both odor experts and in *a priori* untrained individuals who have undergone specific olfactory training in the frame of an experiment, such as repeated exposure to odors or associative learning. Until the 21st century, only the behavioral effects of olfactory training of untrained control individuals had been reported, revealing an improvement of olfactory performance in terms of sensitivity, discrimination, memory, and identification. Behavioral studies of odor experts have been scarce, with inconsistent or inconclusive results. Recently, the development of cerebral imaging techniques has enabled the identification of brain areas and neural networks involved in odor processing, revealing functional and structural modifications as a function of experience. The behavioral approach to odor expertise has also evolved. Researchers have particularly focused on odor mental imagery, which is characteristic of odor experts, because this ability is absent in the average person but is part of a perfumer’s professional practice. This review summarizes behavioral, functional, and structural findings on odor expertise. These data are compared with those obtained using animals subjected to prolonged olfactory exposure or to olfactory-enriched environments and are discussed in the context of functional and structural plasticity.

## INTRODUCTION

Grenouille, who had phenomenal olfactory ability, was able to remember the olfactory imprint of a person and to instantly discern his mood. As a perfumer’s apprentice in 18th-century France, Grenouille attempted to create the ultimate, love-inspiring perfume. However, Grenouille was only a fictional character in a story written by the German writer Süskind (1986). Other testimonies of individuals with a noteworthy sense of smell have been reported in the literature. Bedichek (1960, p. 57), who was a writer, teacher, and naturalist, reported in a posthumously published book that there are “notable noses,” people who are exceptionally sensitive to odors. For instance, he explained that Helen Keller (1908a,b), who described her experience in The Century Magazine, was able to “*recognize an old-fashioned country house because it has several layers of odors, left by a succession of families, of plants, perfumes and draperies.*” Bedichek (1960, p. 57) further highlighted that “*She disentangles and identifies odors by their respective ages, a discrimination I have not found claimed by any nose except that of the bee which one observer declares identifies passage of time by displacement of antennae in flight*.” More recently, Engen (1982), an eminent scientific authority in sensory perception, described an example of experienced noses used in the Vietnam War to detect the whereabouts of machinery and other items. In his famous book, Sachs (1985), a British-American neurologist, also reported the clinical case of a young student, D. Stephen, who experimented with drugs (cocaine, amphetamine). One night, Stephen vividly dreamt that he was a dog, experiencing a world unimaginably rich and significant in smells. On waking, he found that he actually retained this amazingly acute olfactory ability. As emphasized by Engen (1982), one problem with notable noses is that information about them is always anecdotal and is obtained from indirect testimonies, which are not experimentally verifiable. What can we say about the olfactory performances of these noses?

## OLFACTORY PERFORMANCE IN TRAINED INDIVIDUALS AND ODOR EXPERTS

The concept of perceptual learning refers to a phenomenon whereby sensory experience induces changes in behavior and brain function (Gibson, 1991; Goldstone, 1998; Gilbert et al., 2001; Fahle and Poggio, 2002). However, Gawel (1997, p. 268) indicated that the literature does not always clearly delineate what constitutes training and what is experience: “*following training, a panelist can be said to be more experienced, but he can also obtain experience without any formal training.*” Gawel (1997) suggested that, in the first case, better performances result from a uniform and directed program of instruction, whereas in the second case, experience relates to passive exposure to a wide variety of stimuli, which makes them more familiar. He specifies (p. 268) that “*thought may be molded by discussion with others with more or less experience, but always in an unstructured way.*”

In this review, we shall focus on two aspects of perceptual learning by examining data from *a priori* untrained subjects who improved their performance by specific olfactory training (in the frame of an experiment) and from odor experts whose performance is the result of both learning and experience. These experts are mainly perfumers, oenologists, and sommeliers. Surprisingly, most behavioral studies dedicated to evaluating the performance of odor experts have examined wine experts^1^. To the best of our knowledge, only three studies have been devoted to perfumers (Livermore and Laing, 1996; Gilbert et al., 1998; Zarzo and Stanton, 2009). Therefore, when we present expert performances, most of the studies described will concern wine professionals (oenologists and sommeliers). Interestingly, wine discrimination has been used as an example of perceptual learning since the end of the 19th century (James, 1890; Gibson, 1953; Gibson and Gibson, 1955). It is further important to emphasize that wine experts use not only their olfactory system but also their gustatory and trigeminal functions to form a unitary perceptual experience (Small and Prescott, 2005). Wine experts also employ visual perception when identifying a wine (Panghorn et al., 1963; Morrot et al., 2001).

### ODOR SENSITIVITY

In the olfactory domain, the repeated presentation of an odor (within the perithreshold concentration range) in untrained subjects results in the lowering of thresholds and the enhancement of signal detection sensitivity measures (Engen, 1960; Doty et al., 1981; Rabin and Cain, 1986; Dalton et al., 2002). Similar results are observed for volatile substances such as androstenone^2^, for which an individual is conspicuously anosmic but is able to detect with training (Wysocki et al., 1989; Mainland et al., 2002). These data suggest that odor experts who are trained daily can acquire better olfactory sensitivity. However, surprisingly, when the performances of wine experts were compared with those of wine novices or controls, no difference in olfactory sensitivity was revealed for either wine-related components such as tannin or alcohol or non-wine-related components such as *n*-butyl-alcohol (Berg et al., 1955; Bende and Nordin, 1997; Parr et al., 2002; Brand and Brisson, 2012). Bende and Nordin (1997) explained that the non-superiority in detection of wine tasters was due to their professional inexperience with a detection task *per se*. It is also possible that these results were due to the inadequacy of the experimental procedures used in studies.

Several authors state that the plasticity that underpins the emergence of better detection following repeated exposure to odors originates in the central components of the olfactory system, although they do not rule a contribution from peripheral components (Rabin and Cain, 1986; Mainland et al., 2002). In this context, repeated exposure to an odorant (e.g., androstenone, amyl acetate, isovaleric acid, or phenyl ethyl alcohol) can increase olfactory sensitivity to the odorant in mice (Yee and Wysocki, 2001) and rats (Doty and Ferguson-Segall, 1989) and can also increase the sensitivity of the olfactory receptor cells to that odorant in genetically anosmic mice (Wang et al., 1993) and in salmon (Nevitt et al., 1994). Thus, these data provide evidence for stimulus-induced plasticity in sensory receptor cells and suggest that the ability of olfactory cells to exhibit plasticity may be related to their continual turnover (Wang et al., 1993; Huart et al., 2013).

### ODOR DISCRIMINATION

Stimulus “differentiation” also represents an important mechanism of perceptual learning in which experience refines sensory perception through the differentiation of stimulus features, dimensions, or categories (Gibson, 1991; Goldstone, 1998; Schyns et al., 1998). In olfaction, the discrimination task usually consists of comparing two odors in order to determine if they are identical or not^3^. Since it has been claimed that an expert can distinguish as many as 10,000 or even 15,000 odors, not including mixtures (Wright, 1964, 1972), the ability to discriminate between odors could be considered as an area of competence of odor experts. Several studies have shown that wine or beer experts have better discrimination or memory abilities than novices (Walk, 1966; Owen and Machamer, 1979; Peron and Allen, 1988; Solomon, 1990; Bende and Nordin, 1997; Parr et al., 2002; Hughson and Boakes, 2009; Zucco et al., 2011). For instance, Bende and Nordin (1997) reported that sommeliers have greater abilities to discriminate odors of eugenol and citral in a mixture than untrained subjects, although they reported only occasionally experiencing these two odors in their profession. The authors claimed that perceptual learning in odor discrimination can be generalized to other odors as well. Peron and Allen (1988) also demonstrated that novice drinkers of beer improve their ability to discriminate beer flavors with experience.

Rather than evaluating discrimination abilities between two odors, some studies have aimed to determine the maximum number of components that an individual can distinguish within a mixture. Untrained subjects can distinguish only three or four components within a mixture (Laing and Francis, 1989; Schab and Cain, 1992). Using a trained panel of 10 women and an expert panel of 8 male professional perfumers and flavorists, Livermore and Laing (1996) observed that the number of components that experts can discriminate and identify is not higher than that of untrained subjects. Nevertheless, when mixtures of two and three components only were used, experts recorded significantly more hits and fewer false alarms^4^ than did trained non-experts. Livermore and Laing (1996) suggested that the inability of participants to discriminate more than three of four stimuli is a physiologically imposed limit that could be related to the overlap of the odorants’ perceptual or cognitive representations. Thus, when odors are not sufficiently separated in multidimensional perceptual space, the addition of other odorants to the mixture can increase the chance of their representations overlapping, increasing the possibility of perceptual confusion and reducing the ability of the subjects to identify odors. Nevertheless, given that descriptions of wine by sommeliers are usually rich in vocabulary, Hughson and Boakes (2001) suggested that these experts might distinguish more components in a mixture than perfumers or flavorists.

### ODOR MEMORY

**FIGURE 1:**
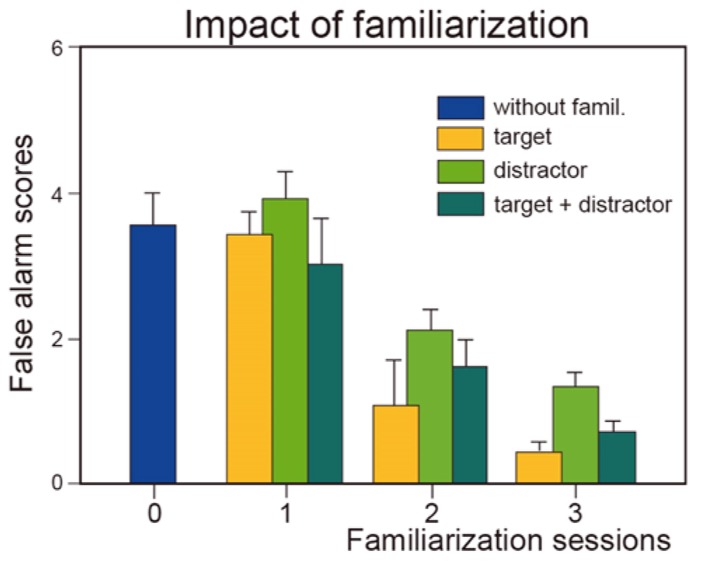
**Effect of familiarization**. Number of incorrect recognitions (false alarm scores) as a function of the number of familiarization sessions (0, 1, 2, and 3) and of the type of odor (target, distractor, or both target and distractor) to which subjects were familiarized. Vertical bars, standard errors of the mean (modified from Jehl et al., 1995).

A wide variety of tests are used to evaluate odor recognition memory (Doty, 1991). One test assesses short-term recognition memory and is similar to the discrimination procedure described above, except that a delay of a few seconds to several tens of seconds separates the two odors of a pair (Engen et al., 1973; Jehl et al., 1994). To our knowledge, only a single study with naïve subjects has investigated the impact of training on odor memory by passive exposure to stimuli (Jehl et al., 1995). The authors demonstrated that familiarization by repeated presentation of target or distractor odors improved discrimination performance by reducing the number of false alarms^5^, that is, incorrect recognition (**Figure 1**). More recently, Hughson and Boakes (2009) evaluated wine drinkers using a different procedure and demonstrated that experience can improve short-term wine recognition (4 min) by passive perceptual learning.

To investigate long-term odor recognition memory, the procedure typically consists of using a set of odors for inspection, followed by the presentation of a second set of odors, including equal numbers of previously presented odors (old) and new odors, in a later testing session (Walk and Johns, 1984). For each item, subjects then indicate whether they have previously smelt the odor or not. Using such a memory test, Rabin and Cain (1984) observed that recognition performances increased with odor familiarity rated at inspection, but they did not specifically examine the influence of repeated presentation of stimuli.

### ODOR IDENTIFICATION

Smell is likely the most difficult sensory modality to verbalize (Wippich et al., 1989). Human beings possess an excellent odor detection and discrimination abilities but typically have great difficulty in identifying specific odorants (Richardson and Zucco, 1989). The fact that there are no specific terms to describe odor and that odors are identified in terms of idiosyncratic personal experience can explain this difficulty. It has been hypothesized that odor information processing shares some of the cortical resources used in language processing and that these two types of processing can interfere with each other (Lorig, 1999).

Correlating with these observations, the human ability to identify and to name^6^ odors is extremely limited (Engen, 1987; Richardson and Zucco, 1989). Estimates vary from approximately 6 to 22 odors when subjects are tested for the first time (Engen, 1960; Sumner, 1962; Desor and Beauchamp, 1974; Lawless and Engen, 1977; Cain, 1979). However, all investigations in naïve subjects have consistently shown that identification performance improves with practice (Desor and Beauchamp, 1974; Cain and Krause, 1979; Cain, 1982). This result is observed as well when subjects must use only labels generated during the first exposure as when they have the option to change labels (Cain, 1979).

### IMPACT OF VERBALIZATION ON OLFACTORY PERFORMANCE

**FIGURE 2:**
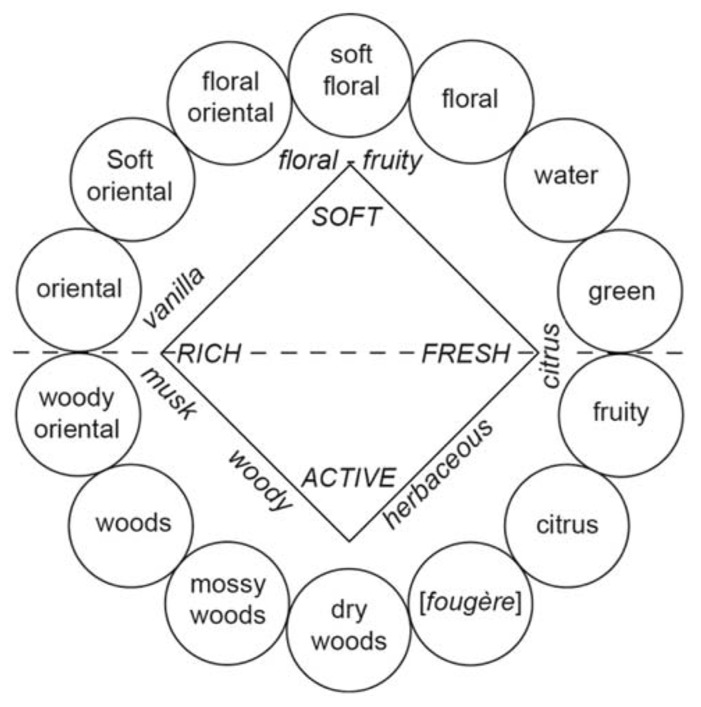
**Fragrance wheel**. Fourteen perfume categories (within circles) are depicted. For the purposes of comparison, the odor effects diagram (inner square, letters in italics) proposed by Calkin and Jellinek (1994) is also illustrated (with permission from Zarzo and Stanton, 2009).

Cain (1979) suggested that experts such as perfumers, flavor chemists, food technologists, and wine tasters must verbalize their olfactory experiences and thus identify odors better than untrained persons. To facilitate the description of complex mixtures of stimuli and the classification of sensations, experts are trained to use descriptors of odors, aromas, and flavors. Accordingly, specific terminologies are employed to describe and classify perfumes (**Figure 2**; Zarzo and Stanton, 2009), wines (Noble et al., 1987), Brandies (Jolly and Hattingh, 2001), or certain alimentary products such as cereals or Cheddar cheese (Chambers and Smith, 1993; Roberts and Vickers, 1994; Drake et al., 2001). Correlatively, it is natural to observe that experts (e.g., trained panelists) better characterize or describe wines (Lawless, 1984; Solomon, 1990; Gawel, 1997; Solomon, 1997; Chollet and Valentin, 2000; Hughson and Boakes, 2001), beers (Clapperton and Piggott, 1979), fishes (Cardello et al., 1982), and perfumes (Lawless, 1988) than non-experts. Consistent with these data, perfumers (or wine professionals) are less prone to classify odors in terms of their hedonic quality than non-experts, suggesting that they are able to discern (or label) perceptual qualities not available to untrained individuals (Yoshida, 1964; Ballester et al., 2008). Chollet and Valentin (2000) suggested that the perceptual representation of wine is similar in experts and novices but the verbalization of this representation varies with the level of expertise. Experts use analytical terms, whereas non-experts use holistic terms (Schab, 1991; Chollet and Valentin, 2000). Gawel (1997) even hypothesized that superior sensorial knowledge in trained panelists not only leads to the search for descriptors but also facilitates the expectation of prototypical characters, which can result in a higher probability of the detection of components.

Discrimination and recognition memory performances of odors and aromas, as described above (see Odor Discrimination and Odor Memory), were evaluated in perceptual terms only. However, except for two studies in which the authors knowingly used unfamiliar odors (Jehl et al., 1994, 1995), semantic impact was likely largely present but not considered in these studies. In addition, it was demonstrated, in an experimental frame, that discrimination and memory performances can partly be improved by verbalization of the stimuli or the knowledge of their names. Such results have been observed in wine experts (Solomon, 1990; Melcher and Schooler, 1996) and in naïve subjects (Lawless and Engen, 1977; Rabin, 1988; Jehl et al., 1997). For instance, Rabin (1988) reported that naïve subjects trained to label specific odors significantly enhanced their ability to discriminate them one day later. According to Rabin (1988, p. 539), “*endowing a layperson with a perfumer’s experience would make subtle mixture components more salient stimuli.*”

In short, it emerges from these data that perceptual (via passive exposure) and cognitive (label learning, development of classification schemas) changes accompany the development of wine expertise (Solomon, 1997; Hughson and Boakes, 2001, 2002; Zucco et al., 2011). However, if perceptual learning of wine, which depends on the frequency and diversity of exposure to stimuli, is rapid and passive, cognitive expertise (semantic) is slower and difficult to develop and requires many years of practice (Zucco et al., 2011). Similar changes are likely associated with the development of expertise in perfumers or flavorists (Jones, 1968; Schab and Cain, 1992). With time, the expert can then acquire perceptual abilities incredibly superior to that of an untrained person (Schab and Cain, 1992).

### ODOR MENTAL IMAGERY

The review of the literature described above shows that it is difficult to propose a test to reveal the higher sensory capacities of odor experts compared to naïve subjects. Data are often conflicting, and it is difficult to decide what is sensory and what is semantic in these tasks. The mental imagery task can satisfy these requirements.

With regards to olfaction, the widespread assertion is that it is very difficult for the average person to mentally imagine odors, in contrast to our ability to mentally imagine images, sounds, or music (Stevenson and Case, 2005; Stevenson et al., 2007). Despite behavioral and psychophysical studies demonstrating the existence of odor imagery (Lyman and McDaniel, 1990; Algom and Cain, 1991; Algom et al., 1993; Carrasco and Ridout, 1993; Ahsen, 1995; Djordjevic et al., 2004a,b, 2005), several authors have even claimed that recalling physically absent odors is not possible (Engen, 1991; Crowder and Schab, 1995; Herz, 2000). However, odor experts do not appear to have difficulty in mentally smelling odors. When perfumers are questioned, they claim that they are quite able to do this and that these images provide the same sensations as the olfactory experiences evoked by odorous stimuli themselves. Gilbert et al. (1998) were the first to investigate olfactory imagery abilities in fragrance experts and to provide evidence that they are better than in non-expert controls. Importantly, they did not observe a difference between the visual mental imagery abilities of the expert and non-expert groups.

## BRAIN REORGANIZATION WITH OLFACTORY PERFORMANCE

The Polish neuroscientist Jerzy Konorski (1948) is regarded as being the first to introduce the term neuroplasticity (also referred to as brain plasticity, cortical plasticity, or cortical re-mapping) to the scientific literature (Jancke, 2009). Konorski presented one of the earliest comprehensive theories of associative learning as a result of long-term neuronal plasticity and also proposed the idea that synapses strengthen with use. The advent of modern brain imaging methods has boosted the study of cortical plasticity in healthy human subjects in the last 20 years (Jancke, 2009). These techniques have enabled the investigation of functional as well as structural plasticity^7^ in experts such as musicians or sportsmen. What about olfactory expertise?

### FUNCTIONAL AND STRUCTURAL DATA IN NON-EXPERTS

A few recent studies suggest that, even in the absence of specific learning, everyday olfactory experience improves olfactory performance and simultaneously shapes olfactory bran regions in the average person (Buschhuter et al., 2008; Frasnelli et al., 2010; Seubert et al., 2013). For instance, the volumes of the olfactory bulb, orbitofrontal cortex (OFC), and insula are positively correlated with the composite measure of olfactory threshold, discrimination, and identification scores (Frasnelli et al., 2010). Moreover, to compensate for their lack of vision, it is well established that blind subjects develop enhanced abilities in the use of their remaining senses. Accordingly, Rombaux et al. (2010) observed that blind subjects have better olfactory performance than sighted control subjects and correlatively have higher olfactory bulb volumes. Congenital or early blind subjects also activate olfactory areas (amygdala, OFC, hippocampus) and occipital areas more strongly than sighted control subjects during an olfactory task (Kupers et al., 2011; Renier et al., 2013), providing evidence that blind individuals undergo adaptive neuroplastic changes.

**FIGURE 3:**
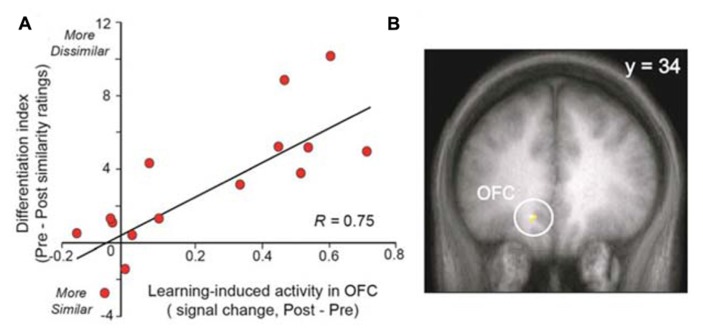
**Experience-induced neural plasticity in the OFC predicts olfactory perceptual learning. (A)** The scatterplot demonstrates a strong correlation between the level of learning-induced OFC signal and the behavioral magnitude of perceptual learning. **(B)** Activation is superimposed on a mean T1-weighted coronal section and displays the area in OFC exhibiting this correlation. OFC, orbitofrontal cortex (modified with permission from Li et al., 2006).

Other studies demonstrate that changes in brain activity can be observed in healthy control subjects after training. Li et al. (2008) demonstrated that odor aversive learning enhances the perceptual discrimination of initially indistinguishable odor enantiomers and that these results parallel the spatial divergence of ensemble activity patterns in the primary olfactory cortex (piriform cortex). These results indicate that aversive learning updates odor quality representations in the piriform cortex or, in other terms, emphasizes a spatial reorganization of odor coding. The same team also demonstrated that prolonged exposure (3.5 min) to a floral-smelling odorant is sufficient to enhance perceptual differentiation of novel odorants that are related in odor quality or functional groups (**Figure 3**; Li et al., 2006). This finding indicates that subjects become floral “experts.” This effect is paralleled by increased responses in both the posterior piriform cortex and the medial OFC. The authors of this older work speculated that this learning-induced plasticity could reflect two neuronal mechanisms: an enlargement of cortical receptive fields that results in the recruitment of more neurons (spatial summation), or, alternatively, a synchronization of neuronal activity (temporal summation; Gilbert et al., 2001).

The results of Li et al. (2006) are echoed by electrophysiological data reported by Wilson (2000, 2003) using anesthetized rats. The authors suggested that perceptual learning via prolonged odorant exposure (habituation) can modify odor-evoked activity in the piriform cortex independently of the responses in the olfactory bulb. These data suggest that adequate sensory experience favors the formation of novel odor representations in the piriform cortex, which could promote olfactory differentiation at both the behavioral (Cleland et al., 2002; Fletcher and Wilson, 2002; Johnson et al., 2002) and neural (Wilson, 2000, 2003) levels.

### FUNCTIONAL AND STRUCTURAL DATA IN ODOR EXPERTS

The first study to investigate brain changes related to odor-taste expertise was reported in 2005. Castriota-Scanderbeg et al. (2005) found that, in contrast to naïve drinkers of wine, who activate the primary gustatory cortex and brain areas implicated in emotional processing (e.g., the amygdala), sommeliers activate more brain regions involved in high-level cognitive processes such as working memory and selection of behavioral strategies (the dorsolateral prefrontal cortex) when they taste wine than when they taste glucose.

The second study was performed in perfumers (Plailly et al., 2012). The authors postulated that, in contrast to laymen, perfumers learn to form olfactory sensory representations through daily practice and extensive training. Because they claim to have the ability to produce perceptual images of smells in the total absence of odorants, we estimated that the ability to form odor mental images is a crucial component of a perfumer’s expertise (Royet et al., 2013). Finally, as for other sensory modalities (Kosslyn et al., 2001), we hypothesized that similar neural networks are activated during mental imagery and the actual perception of odorous sensory stimuli.

**FIGURE 4:**
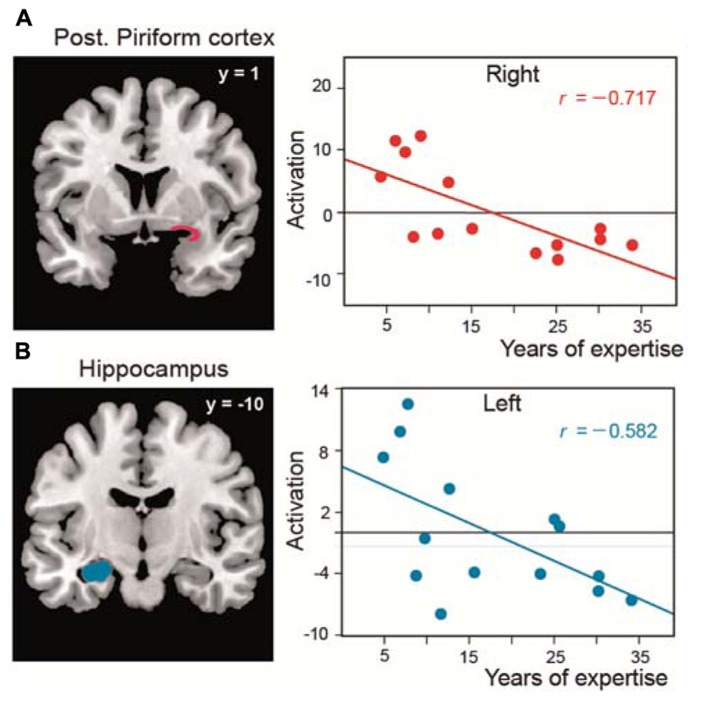
**Functional reorganization in perfumers**. Significant negative correlations between the length of expertise in professional experts and the level of activation (amplitude) in **(A)** the posterior piriform cortices and **(B)** the left hippocampus (modified from Plailly et al., 2012).

As in two studies performed in untrained subjects (Djordjevic et al., 2005; Bensafi et al., 2007), we observed that the piriform cortex is activated when perfumers mentally imagine odors. We further revealed that, during the creation of mental images of odors, expertise influences not only this primary olfactory area but also the OFC and the hippocampus, regions that are involved in memory and the formation of complex sensory associations, respectively. In these areas, the magnitude of activation was negatively correlated with experience: the greater the level of expertise, the lower the activation of these key regions (**Figure 4**). We explained these results in terms of improvements of perceptual capacity and, consequently, gains in performance. Perfumers require less effort to mentally imagine odors than novices. The evocation of mental images is more spontaneous, almost instantaneous, and do not need to rely on high-level cognitive processes to gather information. These abilities, acquired with time and experience, are essential for perfumers because they allow them to devote all of their cognitive resources to the artistic activity that is the creation of novel fragrances.

**FIGURE 5:**
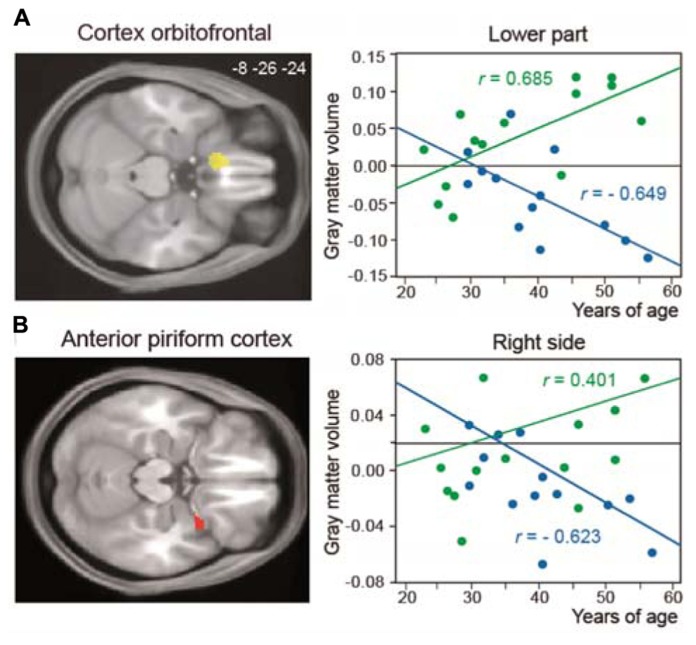
**Structural reorganization in perfumers**. Relationship between structural modifications and years of age. The regression lines between the gray-matter volume and years of age (from 20 to 60 years old) show a positive slope in older experts (OE, green) and a negative slope in older controls (OC, blue) for **(A)** the left GR/MOG and **(B)** the right anterior piriform cortex. GR/MOG, gyrus rectus/medial orbital gyrus (modified from Delon-Martin et al., 2013).

Many studies have shown brain anatomical modifications as a result of learning and training. In experts with enhanced visual, auditory, or motor skills, such as musicians and athletes, greater performances are associated with structural brain changes in modality-specific brain areas. In olfaction, studies indicating structural modifications have only been performed in patients suffering from anosmia, hyposmia, or neurological disease (e.g., Abolmaali et al., 2002; Mueller et al., 2005; Rupp et al., 2005; Rombaux et al., 2006, 2009a,b; Wattendorf et al., 2009; Bitter et al., 2010). Therefore, these studies focus on alterations of olfactory processes associated with atrophy in olfactory-related areas. Recently, we studied structural modifications in the brains of perfumers (Delon-Martin et al., 2013). Using voxel-based morphometry and all possible methodological improvements to reduce false positives, we detected an increase in gray-matter volume in the bilateral gyrus rectus/medial orbital gyrus (GR/MOG), an orbitofrontal area that surrounds the olfactory sulcus, in perfumers. In addition, the gray-matter volumes in the anterior piriform cortex and left GR/MOG were positively correlated with experience in professional perfumers but negatively correlated with age in control subjects (**Figure 5**), suggesting that training counteracts the effects of aging.

Our data are the first to demonstrate the functional and structural impact of long-term odor training. What characterizes odor experts compared with other types of experts? Professional musicians practice several hours a day; their practice begins early in life and continues intensively throughout their lives. Sportsmen such as gymnasts or swimmers also begin early in life, but their careers end more rapidly than those of musicians, at approximately 30–35 years of age, when their physical performance does not allow them to be competitive. In contrast to musicians and sportsmen, odor experts such as perfumers and flavorists begin their training only in early adulthood, at the beginning of their working life or when they join a specialized school. They then live in an enriched olfactory environment in which they learn to characterize and recognize numerous stimuli daily and to learn to discriminate minute differences between odors. They can continue their training into old age. Olfactory performance is usually reported to decrease with age in the layman (e.g., Doty et al., 1984; Stevens et al., 1990; Murphy et al., 1991), and these deficits are partly due to both degenerative processes within the olfactory epithelium (Doty et al., 1984; Welge-Lussen, 2009) and changes in central olfactory structures (e.g., Tomlinson and Henderson, 1976). However, our functional and structural data demonstrate that perfumers can improve their performance throughout their lives and that intensive olfactory training can also counteract the effects of age. The volume of several brain regions involved in odor processing increases in perfumers but decreases in laymen. Thus, the metaphor “*use it or lose it*” used by Jancke (2009, p. 535) in reference to brain plasticity can also be applied to the olfactory modality. Furthermore, even if a peripheral dysfunction is observed in elderly odor experts, our findings further suggest that elderly perfumers would still be able to mentally imagine perfumes, just as deaf professional musicians are still able to continue to compose and conduct by mentally imagining music.

### NEURONAL AND CELLULAR MECHANISMS RELATED TO OLFACTORY LEARNING

In the frame of our functional study in which perfumers were asked to generate mental images of odors (Plailly et al., 2012), a decrease in the amplitude of brain activation with the level of expertise could be due to greater selectivity of neurons resulting from the decorrelation of neuronal activity (Gilbert et al., 2001). Similar mechanisms have been observed in the antennal lobe of honeybees that are trained on one odorant. The sensorial representation of that odorant becomes smaller, more compact, and non-overlapping with representations of other odorants (Faber et al., 1999). This effect has also been observed in rats that are trained to discriminate highly overlapping odorous mixtures (Chapuis and Wilson, 2012).

**FIGURE 6:**
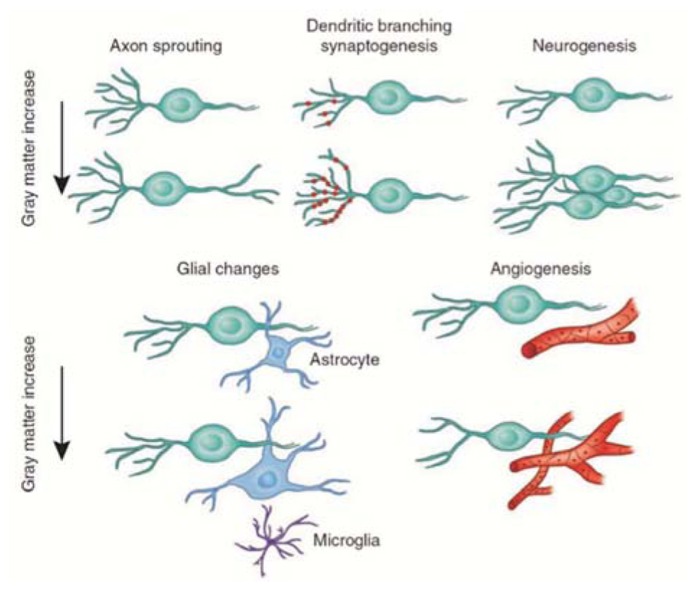
**Candidate cellular mechanisms for gray matter plasticity**. Cellular events in gray matter regions underlying changes detected by magnetic resonance imaging during learning include axon sprouting, dendritic branching, and synaptogenesis, neurogenesis, changes in glial number and morphology, and angiogenesis (image courtesy of Marina Corral; modified with permission from Zatorre et al., 2012).

The nature of the cellular events that underlie structural changes in the human brain is still unknown (May, 2011), although it is widely assumed that gray matter loss in neurodegeneration corresponds to neural loss (Baron et al., 2001; Thieben et al., 2002). Several mechanisms have been proposed to explain increases in gray matter: neurogenesis, gliogenesis, synaptogenesis, and vascular changes (**Figure 6**; Zatorre et al., 2012). We will discuss only the two main mechanisms related to neuronal activity-dependent changes in gray matter.

First, gray matter increases can be explained by fast morphological changes in the intracortical axonal architecture, including the formation of new connections by dendritic spine growth (i.e., synaptogenesis) and changes in the strength of existing connections (Trachtenberg et al., 2002). These changes have been implicated in experience-related morphological modifications in the rat hippocampus (Moser et al., 1994; Geinisman et al., 2000; O’Malley et al., 2000) and have been suggested as a mechanism (long-term potentiation) underlying long-term memory (Bliss and Collingridge, 1993; Luscher et al., 2000). A 3-day olfactory learning in rats is accompanied by a dendritic spine density increase (15%) along apical dendrites of pyramidal neurons in the piriform cortex, suggesting an increased number of excitatory synapses (Knafo et al., 2001). As activity-induced dendritic morphogenesis in the hippocampus can occur within tens of minutes (Maletic-Savatic et al., 1999), the perceptual learning observed by Li et al. (2006) could be associated with such modifications.

Second, gray matter increases can be related to slow mechanisms, such as adult neurogenesis, which has been reported in the olfactory bulbs of rodents and primates, including humans (Bonfanti and Peretto, 2011; Curtis et al., 2011; Ming and Song, 2011; Huart et al., 2013; Lazarov and Marr, 2013). Although the functional impact of the addition of new olfactory neurons to mature circuits remains an outstanding question, many recent investigations have highlighted the role of network activity in shaping ongoing neurogenesis and, in turn, how the integration of new neurons refines pre-existing network functions and, consequently, olfactory behavior. To date, olfactory adult neurogenesis was associated with an improvement in short-term olfactory memory when mice were exposed daily to a novel but not familiar enriched olfactory environment (Rochefort et al., 2002; Bovetti et al., 2009; Veyrac et al., 2009). It was also demonstrated that olfactory perceptual learning both increases and requires adult neurogenesis (Moreno et al., 2009). Interestingly, constitutive neurogenesis has been described in the adult piriform cortex in several mammalian species (Bernier et al., 2002; Shapiro et al., 2007). Here, we suggest that the gray matter volume increase in the piriform cortex of perfumers could result from a fast remodeling of the intracortical neuronal network, but genesis of new neurons in this brain area cannot be excluded.

## CONCLUSION

This review of the literature presents the findings of studies in which odor experts were subjects. In contrast to other domains of expertise, odor expertise has been rarely studied (Ericsson and Lehmann, 1996; Vicente and Wang, 1998; De Beni et al., 2007). In 1998, Vicente and Wang wrote that there were at least 51 studies of the effects of expertise in at least 19 different domains, including music (e.g., piano), sport (e.g., skating, baseball), games (e.g., bridge, go, chess), computer programming, medical diagnosis, maps, algebra, and circuit diagrams. The model of expertise research is the chess player because experts can reach very high levels of competence and the ability of participants is measurable and can be rated in a laboratory (De Beni et al., 2007). In all cases, studies of expertise emphasize the role of long-term working memory on performance (Ericsson and Kintsch, 1995) and highlight that “*memory recall performance on meaningful stimuli has almost always been found to be correlated with domain expertise*” (Vicente, 1988; Vicente and Wang, 1998, p. 33).

The extremely high performance of experts begs the fundamental question of whether their faculties are innate or acquired with training. In 1869, Francis Galton claimed that, because the limits on height and body size are genetically determined, innate mechanisms must also determine mental capacities (see Galton, 1979). Ericsson and Lehmann (1996) suggested that the influence of innate, domain-specific basic capacities (talent) on expert performance is small, possibly even negligible. However, more recent studies indicate that characteristics that distinguish experts from naïve subjects are mainly the result of adaptation. High expertise is typically associated with prolonged and maintained practice lasting many years and involving daily exercises (De Beni et al., 2007). The apparent emergence of early talent then depends on factors “*such as motivation, parental support, and access to the best training environments and teachers”* (Ericsson et al., 2009, p. 199).

In the context of odor experts, it is likely that expertise is acquired with training and experience rather than acquired innately, thus confirming a previous report that the notable nose is bred rather than born (Bedichek, 1960, p. 61; Engen, 1982, p. 5). Our work in cerebral imaging has led us to the same conclusions. Olfactory mental imagery capacities develop with practice and do not result from innate skill (Plailly et al., 2012). The structural modifications observed in the brain after intensive practice of an activity are not stable and rapidly disappear when this activity stops (Jancke, 2009). However, an exception that deserves to be noted is the case of synesthetes, who possess faculties to perceive a given sensory stimulus via another or several other sensory modalities. Synesthesia is a rare phenomenon that can have a genetic origin, which could explain the exceptional performances of experts such as mental calculators. Although relatively less frequent, examples of synesthesia involving olfactory sensation have been described in the literature (Day, 2005).

## Conflict of Interest Statement

The authors declare that the research was conducted in the absence of any commercial or financial relationships that could be construed as a potential conflict of interest.

## Footnotes

1 We identified approximately 50 studies devoted to wine expertise (without taking into account expertise of other types of alcohol such as beer or brandy). This number is not huge but is much higher than the three behavioral studies that have been devoted to perfumers. Whereas the number of perfumers in the world is approximately 500 (120 in France and Switzerland), the number of oenologists (without sommeliers) can be estimated at more than 150,000 (of which 9,500 live in France) in 44 wine-producing countries.

2 Androstenone is a pheromone that has been identified in pigs. Although this steroid is also found in sweat and urine of both human male and female, and that gender-specific differences in olfactory sensitivity to this odor have been demonstrated (see, e.g., Dalton etal., 2002), it has not yet been recognized as being a human pheromone. Androstadienone, that is a compound closely-related to androstenone, has also been suggested to be a human pheromonal substance.

3 Other types of discrimination tasks are used, such as the triangle test, in which three samples, two of which are identical, are presented to participants. The task consists of determining which stimulus is different (Amerine etal., 1965). Another task asks subjects to rank samples along a sensory dimension. In the case of wine, the sensory dimension can be attributes of odor (e.g., alcohol, fruit) or taste such as sugared or astringency (produced by tannin; Solomon, 1990).

4 In such a discrimination task, a hit is defined when the subject correctly identifies a component that is present; a false alarm is defined when the subject incorrectly identifies a component as being present.

5 In the short-term recognition task, the subject must indicate whether the two odors of a pair are identical or different. A hit is defined when the two odors are identical and are so declared by the subject. A false alarm is defined when the two odors are different but are declared as identical by the subject.

6 In a typical multiple-choice identification test, the subject has a list of labels when the olfactory stimulus is presented. One of the labels is veridical (e.g., strawberry). A second label is an alternative name and evokes a similar odor (a near miss, such as raspberry). Other names are more distinct alternatives (far misses, such as tar). The number of names can vary from three to four to several dozen. In a naming test, only the odor is presented to the subject. This test is therefore more difficult than the multiple-choice test. The results can be analyzed in terms of response accuracy (veridical label, near and far misses; see, e.g., Rabin and Cain, 1984; Lyman and McDaniel, 1986).

7 The concept of “functional brain plasticity” refers to modifications of brain activity, whereas “structural brain plasticity” refers to changes at the anatomical level.
